# P-1669. Effect of Antimicrobial Stewardship Program on Antibiotic Prescribing for Veterans Affairs Nursing Homes

**DOI:** 10.1093/ofid/ofae631.1835

**Published:** 2025-01-29

**Authors:** Matthew Jacob, Morgan Froehlich, Debra Belanger, George Psevdos

**Affiliations:** Stony Brook University Hospital, Stony Brook, New York; Northport VAMC, Northport, New York; Northport VAMC, Northport, New York; Northport VA Medical Center, Northport, New York

## Abstract

**Background:**

The CDC strongly recommends nursing homes take resolute steps to improve antibiotic prescribing practices and reduce inappropriate use. This is because studies have shown that up to 70% residents of US nursing homes receive 1 or more courses of antibiotics in a year while 75% of these prescriptions may be unnecessary or inappropriate. Antibiotic stewardship programs (ASP) are committed to optimize treatment of infections and reduce adverse events by close monitoring, education, and preventing unsuitable use. We analyzed the effect of our ASP over antibiotic prescribing of four Veterans Affairs (VA) community living centers (CLC).

Table 1.

**Figure:**
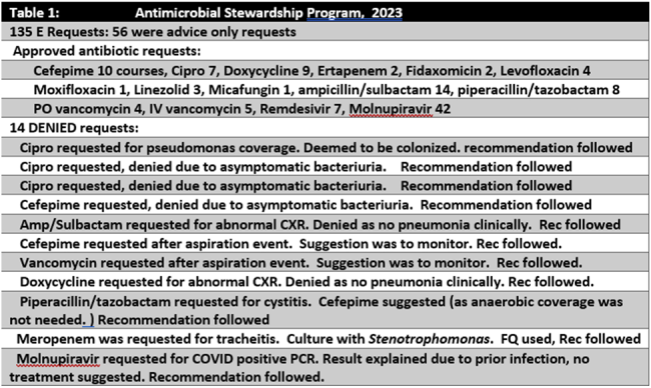

**Methods:**

Retrospective review of ASP notes in the year 2023- the inaugural year for ASP reviewing CLCs- addressing requests for restricted antibiotics over 4 CLCs at Northport VA campus. The 4 CLCs are structured nursing homes with a 139 total bed capacity with two long term residential care units, one mental health, and one combined subacute rehab/hospice. We compared antibiotic use as days of therapy (DOT)/1000 days, for total antibiotics use, and analyzing cephalosporins, fluoroquinolones anti-MRSA, anti-MSSA, for years of pandemic 2020-2022 vs. 2023.

Figure 1a

**Figure:**
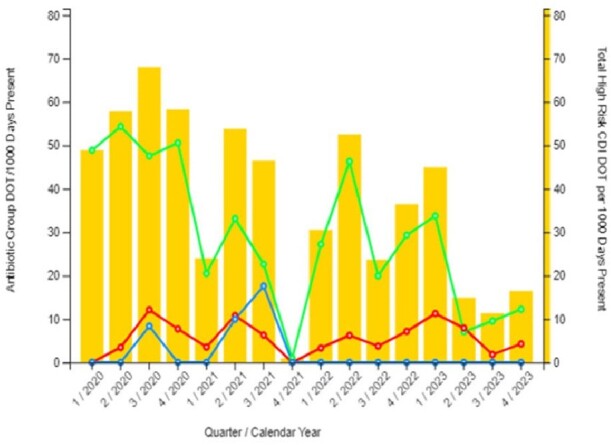

**Results:**

Comparing 2020-22 vs. 2023 there was a decrease in all antimicrobial use in the CLCs, from 142 DOT/1000 days to 88. In 2023 the ASP team reviewed 135 CLC requests for restricted antibiotics, and 56 E-advice requests for guidance. 10% of the requests were denied. See table 1. The most common request for E- advice was whether to treat asymptomatic bacteriuria with/or without presence of urinary catheter. Comparing 2020-22 vs. 2023 there was a decrease in cephalosporins use by DOT/1000 days, 29.3 vs. 10.8, P: 0.09. There was no difference in Fluoroquinolones 2.0 vs. 6.0 P: 0.953, anti-MRSA antibiotics, 7.02 vs. 4.04 P:0.348, anti MSSA 0.67 vs. 0.8 P: 0.574. See figures 1a/1b. Figure 2 shows a downward trend of C. *difficile* infections in our CLCs.

Figure 1b

**Figure:**
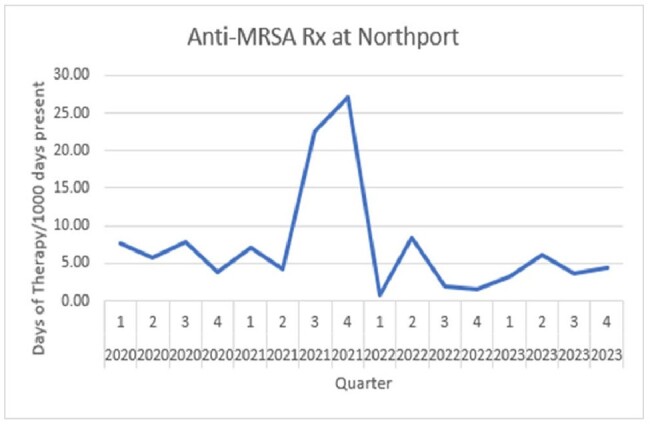

**Conclusion:**

Our ASP was overall successful in decreasing antibiotic use in our CLCs, notably for cephalosporins, while keeping FQ and other antibiotics use low. We were successful in education and preventing antibiotics especially for asymptomatic bacteriuria. An equally important achievement for our program was the decreasing rate of *C. difficile* infections.

Figure 2

**Figure:**
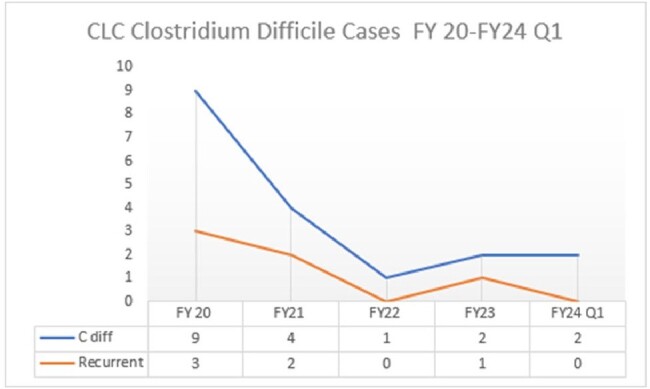

**Disclosures:**

**All Authors**: No reported disclosures

